# Development of two novel high-throughput assays to quantify ubiquitylated proteins in cell lysates: application to screening of new anti-malarials

**DOI:** 10.1186/s12936-015-0708-1

**Published:** 2015-05-14

**Authors:** Lydia Mata-Cantero, Concepción Cid, Maria G Gomez-Lorenzo, Wendy Xolalpa, Fabienne Aillet, J Julio Martín, Manuel S Rodriguez

**Affiliations:** Present address: Tres Cantos Medicines Development Campus, Diseases of the Developing World, GlaxoSmithKline, Severo Ochoa 2, Tres Cantos, 28760, Madrid Spain; Centro de Investigación Básica, GlaxoSmithKline, Santiago Grisolía 4, 28760 Tres Cantos, Madrid Spain; Ubiquitylation and Cancer Molecular Biology, Inbiomed, Mikeletegi 81, 20009 San Sebastian, Spain

**Keywords:** *Plasmodium falciparum*, Malaria, Ubiquitin proteasome system, High-throughput screening, Tandem ubiquitin binding entities, Cellular assay, Drug discovery

## Abstract

**Background:**

The ubiquitin proteasome system (UPS) is one of the main proteolytical pathways in eukaryotic cells and plays an essential role in key cellular processes such as cell cycle, stress response, signal transduction, and transcriptional regulation. Many components of this pathway have been implicated in diverse pathologies including cancer, neurodegeneration and infectious diseases, such as malaria. The success of proteasome inhibitors in clinical trials underlines the potential of the UPS in drug discovery.

**Methods:**

*Plasmodium falciparum*, the malaria causative pathogen, has been used to develop two assays that allow the quantification of the parasite protein ubiquitylation levels in a high-throughput format that can be used to find new UPS inhibitors.

**Results:**

In both assays tandem ubiquitin binding entities (TUBEs), also known as ubiquitin traps, have been used to capture ubiquitylated proteins from cell lysates. The primary assay is based on AlphaLISA technology, and the orthogonal secondary assay relies on a dissociation-enhanced lanthanide fluorescent immunoassay (DELFIA) system. A panel of well-known proteasome inhibitors has been used to validate both technologies. An excellent correlation was obtained between these biochemical assays and the standard whole cell assay that measures parasite growth inhibition.

**Conclusions:**

The two assays presented can be used in a high-throughput format to find new UPS inhibitors for *P. falciparum* and could help to identify new targets within this system. This methodology is also applicable to other cellular contexts or pathologies.

## Background

*Plasmodium falciparum* is responsible for the most severe form of human malaria. Spreading resistance to current treatments urges the need for new medicines with novel modes of action. After decades pursuing target-based programmes, the focus now is on phenotypic screenings, as the knowledge of the biology of the parasite is limited and, at least in theory, inhibitors of any essential pathway of the parasite could be found. One such essential pathway is the ubiquitin proteasome system (UPS) which consists in a covalent, post-translational modification that orchestrates the function and turnover of numerous cellular proteins and regulates many essential cellular processes, such as cell cycle progression, transcription, endocytosis, DNA repair, apoptosis, signal transduction, differentiation, cellular stress, and protein trafficking. Alterations of the UPS have been implicated in a large number of diseases, including many cancer types, neurodegenerative and immunological disorders, and also infectious diseases. Therefore, the UPS has become one of the most promising targets for drug development [1].

The UPS consists of multiple enzymes and cofactors that regulate the attachment/detachment of ubiquitin to target proteins before being exposed to the 26S proteasome. Ubiquitin is a highly conserved protein among eukaryotes showing only few amino acid differences between mammalian and yeast versions [2]. The protein modification process, also known as conjugation, requires three sequential steps that start with the activation of the C-terminal glycine residue of the ubiquitin by an ubiquitin-activating enzyme (E1), which forms a thiolester linkage with ubiquitin. This activated ubiquitin is transferred to an ubiquitin-conjugating enzyme (E2) and then to an ubiquitin-ligase (E3) that specifically interacts with the protein substrate. The C-terminal glycine of ubiquitin is attached to an amino group of a lysine present in the substrate. Additionally, ubiquitin has seven active lysines that can accept ubiquitin moieties generating different types of polyubiquitin chains. Some polyubiquitin chains have been associated with specific cellular functions. For example, K63 chains that activate signalling cascades or K48 and K11 chains that are linked to protein degradation by the proteasome. The process can be reverted by the action of deubiquitylating enzymes (DUBs) that are responsible for the dynamic equilibrium of the system.

The 26S proteasome is a multi-sub-unit complex formed by a 20S core particle, responsible for the catalytic activity, and by regulatory 19S particles flanking each end of the core to control the entry of ubiquitylated proteins. The 20S core consists of four heptameric rings, the two outer rings are formed by alpha sub-units and the two inner rings are composed of beta sub-units. β1, β2 and β5 sub-units are responsible for the peptidyl-glutamyl peptide-hydrolyzing (PHGH), the trypsin and the chymotrypsin-like activities of the proteasome, respectively [3].

Several UPS components have been considered as druggable targets since some of them are directly involved in different human diseases. Proteasome was the first successful target within the UPS. The proteasome inhibitor, bortezomib reached clinical phases for the treatment of various types of cancer. Since its approval for the treatment of multiple myeloma and mantle cell lymphoma in 2003, another four inhibitors of the UPS are in clinical trials and seven more are in preclinical studies [4-7]. The efficacy and limited toxicity of these inhibitors are based on the fact that rapidly dividing cancer cells are more sensitive than non-dividing ones suggesting that very active processes will be better blocked by UPS inhibitors.

**Figure 1:**
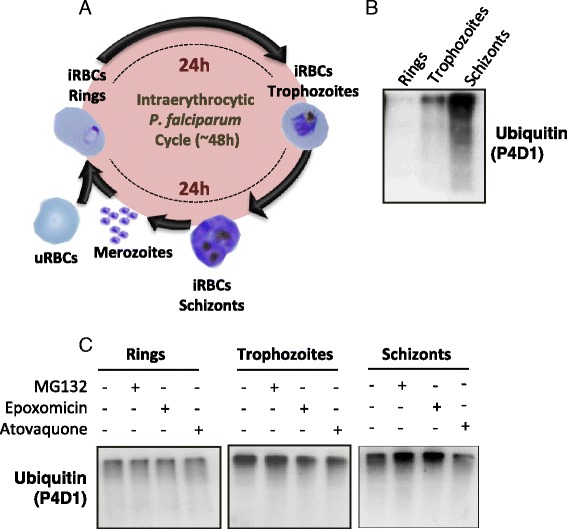
Analysis of ubiquitylated proteins along *Plasmodium falciparum* intra-erythrocytic cycle. **A**. Schematic representation of the parasite cycle in blood stages. It starts when merozoites invade uRBC resulting in the first phase called ring stage. After 24 hrs, the parasite enters in a phase with high metabolic activity, the trophozoite. A few hours later, at the schizont stage, the parasite produces merozoites that will rupture the RBC membrane, looking for new uRBCs to invade and start a new cycle. **B**. Western blot analysis with P4D1 antibody shows basal levels of ubiquitylated proteins in rings, trophozoites and schizonts. **C**. Effect of proteasome inhibitors MG132 (1.15 μM) and epoxomicin (0.26 μM), on the levels of ubiquitylated proteins from rings, trophozoites and schizonts. Atovaquone at 0.02 μM was used as negative control.

*Plasmodium falciparum* divides rapidly during its intra-erythrocytic cycle (see Figure 1A), fulfilling the criteria to be targeted by a UPS inhibitor. Moreover, multiple evidence indicates that the UPS is involved in the parasite cell cycle progression and protein quality control [8,9]. Even though there is a conserved sequence homology between the parasite and human proteasome proteins, there is space for selectivity not only in the proteasome but also at specific components of the UPS such as E3 ligases and DUBs [10-12]. Targeting *P. falciparum* UPS can provide novel modes of action to overcome the emerging resistance to current treatments, as already demonstrated *in vitro* [13]. Indeed, proteasome inhibitors can efficiently inhibit *P. falciparum*-resistant strains at various stages of the cycle in the nanomolar range and with limited toxicity to humans [13-15]. New inhibitors targeting specifically the parasite UPS are desirable [16] to reduce possible side effects.

Thus, the aim is to develop assays that can detect inhibitors of any of the steps involved in UPS, with a high throughput. Even though there are some assays published that measure UPS activity, they are based on quantifying proteasome activity, either biochemically or in cell-based assays using engineered strains. These assays give a large number of false positives and negatives and can be biased by a different homeostasis of the modified cell [17-21]. The effect of compounds on the whole UPS can also be assessed by measuring the total level of ubiquitylated proteins that are present in cell lysates after treatment. Proteasome inhibition causes an accumulation of ubiquitylated proteins that can be analysed by Western blot [22]. However this method is costly and time consuming and hence is not suitable for high-throughput screening (HTS). Assays with higher throughput using TR-FRET and AlphaLISA in combination [23,24] with tandem ubiquitin binding entities (TUBEs) [25], have also been published, but they are target-based approaches that make use of recombinant proteins. Moreover, they are limited to a precise activity within the UPS, therefore previous knowledge about the target is required.

Here, the development of two cellular assays to quantify the total pool of ubiquitylated proteins in *P. falciparum* cell lysates is presented, which can be used as primary and secondary assays in a HTS campaign to find inhibitors of the UPS as a whole, that is, inhibitors of any step that can significantly alter the level of ubiquitylated proteins*.* These assays are based on the use of TUBEs, which are tandem ubiquitin-associated domains (UBA) fused to a glutathione S-transferase (GST) or biotin tag that allow the purification of captured ubiquitin conjugates in native conditions after cell lysis. The tandem disposition of UBA domains increases the affinity for ubiquitin chains and also protects ubiquitylated proteins for DUBs-mediated deconjugation and proteasomal degradation [25-27].

The first assay developed is a homogeneous assay based on the AlphaLISA technology that can be used as a primary test to screen any compound collection to identify molecules that putatively alter the levels of ubiquitylated proteins. The second technique is a heterogeneous dissociation-enhanced lanthanide fluorescent immunoassay (DELFIA) assay that can be useful to confirm the positive compounds found in the primary assay, and hence discard false-positive compounds associated with AlphaLISA technology. This heterogeneous assay requires washing steps, thus increasing the time of manipulation and results deviation and has higher associated costs when used for HTS. However, the advantage is the reduction of background interference by compounds and biological components of the assay. The DELFIA assay could also be used independently as a primary assay during hit optimization process to assess the potency of compounds in dose–response experiments. Assays described here have been validated using a panel of well-known proteasome inhibitors and could be used to quantify the UPS activity in cells or to run HTS campaigns in order to find selective inhibitors of this pathway. These universal methods have been set up using *P. falciparum*-infected red blood cells (iRBCs) as the biological system to identify compounds that alter UPS, but conditions could be adapted easily to other cell types and pathologies or to study and/or find specific inhibitors of particular components of the UPS.

## Methods

### Reagents

Hypoxanthine, sorbitol, percoll, MG132, Epoxomicin, Lactacystin, Clasto-lactacystin β-lactone, Gliotoxin, MG115, EDTA, sodium phosphate dibasic, monosodium phosphate, Tris HCl, sodium chloride, Tween-20, sodium pyrophosphate, glycerol-2-phosphate, saponin, bovine serum albumin (BSA), dithiothreitol (DTT) and phenylmethylsulphonyl fluoride (PMSF) were purchased from Sigma. Glutathione acceptor beads, protein A donor beads, DELFIA enhancement solution and DELFIA secondary antibody (Eu-N1 rabbit Anti-mouse-IgG) came from Perkin Elmer. NP-40 was purchased from Calbiochem, sodium fluoride from Panreac, antibody anti-ubiquitin P4D1 from Santacruz, deubiquitylases inhibitor PR-619 came from Merck, complete mini EDTA protease inhibitor cocktail from Roche, antibody anti-ubiquitin FK2 from Enzo, biotin-TUBEs from Life sensor, RPMI 1640 medium from Gibco, AlbuMAX II from Invitrogen, bortezomib from Selleckchem, enhanced chemiluminescence (ECL) from GE Healthcare and PBS from Oxoid. Atovaquone was prepared in house.

### *Plasmodium falciparum* cultures

All experiments were performed using *P. falciparum* strain 3D7A obtained from the Malaria Research and Reference Reagent Resource (MR4) [28]. Parasites were maintained in culture in T150 flasks in a 5% CO_2_, 90% N_2_ and 5% O_2_ atmosphere, using RPMI 1640 medium supplemented with 5% AlbuMAX II, 150 μM hypoxanthine and fresh red blood cells (uRBCs) according to the methods previously described [29,30]. uRBCs were obtained from the Spanish Red Cross Blood Bank.

### GST-TUBEs expression and purification

TUBEs tagged with GST were expressed in *Escherichia coli* C41 (DE3) as previously reported [25], although they are also available from Lifesensors. Expression of TUBEs was induced with 1 mM IPTG for 6 hrs at 20°C. Bacteria were lysed by sonication in PBS with 1% triton X-100 and 2 mM benzamidine and lysates were clarified by centrifugation at 20,000 rpm for 2 hrs at 4°C. TUBEs were purified by standard glutathione agarose beads following manufacturer instructions. Briefly, lysate was incubated with glutathione agarose beads for 2 hrs at 4°C and then these beads were washed with 1% triton X-100 in PBS. Recombinant TUBEs were eluted with 10 mM glutathione in 50 mM Tris–HCl pH 9.5. After a buffer exchange to PBS (Amicon ultra centrifugal filter cut off 3 kDa) protein concentration was determined by absorbance at 280 nm.

### Western blot analysis

*Plasmodium falciparum* cultures were synchronized with 5% w/v sorbitol enriching iRBCs in ring stage, and maintained for three days at 0.8% haematocrit and 2% parasitaemia as previously described [30]. Haematocrit is defined as the volume percentage of RBCs and parasitaemia is the percentage of iRBCs in the culture. Then, a new synchronization cycle was done through a 70% percoll gradient (v/v) isolating iRBCs highly synchronized in schizont stage [30]. Culture was adjusted to 5% parasitaemia and 1% haematocrit with fresh uRBCs and grown for an extra day. Then, iRBCs were treated with proteasome inhibitors MG132 (1.15 μM) or Epoxomicin (0.26 μM) and incubated for 1 hr at 37°C at 20 times the IC50 that causes the parasite growth inhibition. Samples were taken at three different time points; time zero (rings), 14 hrs (trophozoites) and 24 hrs (schizonts). Atovaquone, an anti-malarial drug, was used at 0.02 μM as a negative control since it does not target the UPS system. Cultures, without any treatment, were also taken to assess the basal levels of ubiquitylated proteins. *Plasmodium falciparum* cultures were pelleted by centrifugation at 600 g for 5 mins and frozen at −80°C for at least 24 hrs. Then, pellets containing the parasites were treated with 0.1% w/v saponin in PBS for 5 min at 4°C. Cells were centrifuged at 3,500 rpm for 5 min and washed three times with cold PBS to remove haemoglobin until the supernatant was clear. The cell pellets corresponding to purified parasites were resuspended in lysis buffer (50 mM NaF, 5 mM tetra-sodium pyrophosphate, 10 mM β-glyceropyrophosphate, 1% NP-40, 2 mM EDTA, 20 mM Na_2_HPO_4_, 20 mM NaH_2_PO_4_, 1 mM PMSF and complete mini EDTA protease inhibitor cocktail) and lysed with two cycles of sonication (10 sec each). Lysates were cleared by centrifugation for 10 min at 14,000 rpm and 4°C and supernatant collected. Protein amount was quantified by Bradford. For each condition, equal amount of proteins were separated by SDS-PAGE. Proteins were then transferred onto polyvinylidene difluoride (PVDF) membranes (Roche) and incubated with anti-ubiquitin P4D1 antibody diluted 1:1,000 to analyse ubiquitylated proteins.

### *Plasmodium falciparum* extracts for assay development

To set up the assays, *P. falciparum* parasites at schizont stage were isolated from RBCs to remove haemoglobin which causes interference in the AlphaLISA technology. *Plasmodium falciparum* cultures were synchronized with sorbitol and percoll as described in the previous section. Cells were grown for two days at 1% haematocrit and 5% parasitaemia. Then, cultures enriched with schizonts at 10% parasitaemia were treated for 1 hr with the proteasome inhibitor MG132 at 1.15 μM or kept without treatment as basal control, depending on each experiment. iRBCs were centrifuged at 600 g for 5 min and frozen at −80°C. Parasites were extracted with 0.1% w/v saponin, washed three times in PBS and then lysed by sonication in lysis buffer (50 mM Tris pH 7.5, 5 mM EDTA, 150 mM NaCl 1% Tween 20, complete mini-EDTA free anti-proteases, 1 mM PMSF, 50 μM deubiquitylase inhibitor PR-619 (DUBI), a cell-permeable broad spectrum inhibitor that does not affect the proteasome activity and it is used to protect against deubiquitylation processes in the lysis moment) as described above, obtaining a clear parasite protein lysate without haemoglobin. Lysates were then dispensed in 384-or 1,536-well plates to assess levels of ubiquitylated proteins by AlphaLISA or DELFIA assays under different conditions (see Results).

### Purification of iRBCs with schizonts and treatment with compounds

The developed assays were designed to use *in vitro P. falciparum* iRBCs highly synchronized in schizonts following the protocol previously described [30]. Briefly, *P. falciparum* cultures were synchronized using sorbitol and percoll treatments as described above. Cultures adjusted to 5% parasitaemia and 1% haematocrit after percoll synchronization were grown for 48 hrs and then passed through magnetic MACs columns type CS (Miltenyi biotec). These columns only retain RBCs that are infected with mature forms of *P. falciparum*, enriching the eluate with a parasitaemia of iRBCs in schizont stage up to 98%. Cells were pelleted by centrifugation at 600 g for 5 min and adjusted with supplemented RPMI 1640 medium to 0.4% haematocrit for AlphaLISA and 0.2% haematocrit for DELFIA assay. Cultures were kept in the incubator at 37°C for 1 hr and then dispensed into plates containing proteasome inhibitors: 2 μL in 1,536-well white plates (Greiner) containing 20 nL of compound for AlphaLISA and 20 μL in 384-, flat-bottom, black plates containing 200 nL of compound for DELFIA assay. Proteasome inhibitors were dissolved in 100% DMSO, being 1% the final DMSO concentration in the assays. Plates with iRBCs and compounds were incubated at 37°C for 1 hr and then frozen at −80°C.

### TUBE-AlphaLISA assay

Assay was set up in 1,536-well white plate, in which 2 μL of each reagent were added per well and step, reaching all together a final volume of 10 μL. Frozen plates containing 2 μL of iRBCs and 20 nL of compounds (as described in the previous section) were thawed, to avoid deconjugation processes, in presence of 2 μL of lysis buffer containing TUBEs (50 mM Tris pH 7.5, 5 mM EDTA, 150 mM NaCl, 1% Tween 20, 50 μM DUBI, complete mini-EDTA free anti-proteases, 1 mM PMSF and 25 μg/mL GST-TUBEs). Lysis was performed by three fast freeze/thaw cycles (10 min each) and then the plates were kept at room temperature for 1 hr to enable the efficient capture of ubiquitylated proteins by the GST-TUBEs. Two μL of AlphaLISA GSH acceptor beads at 0.1 μg/μL diluted in AlphaLISA assay buffer (100 mM sodium phosphate buffer pH 7, 0.1% BSA) with 7.5 mM DTT were added. Plates were sealed and incubated for 4 hrs at 4°C to allow the binding of GSH beads with the GST-TUBEs. Then, 2 μL of anti-ubiquitin FK2 antibody (diluted 1:80 in AlphaLISA assay buffer) was dispensed and incubated for 1 hr at 4°C in darkness. Finally, 2 μL of protein A donor beads at 0.2 μg/μL (in assay buffer) were added and plates were incubated in darkness at 4°C overnight, enabling the binding of the FK2 antibody and the donor beads. The levels of ubiquitylated proteins were determined by measuring the light emission at 615 nm in the Envision multilabel reader (Perkin Elmer) using the AlphaLISA protocol.

### TUBE-DELFIA assay

In a typical assay, the addition of each reagent was preceded by six washing steps with 100 μL of TBS-Tween (50 mM Tris HCl, 2.7 mM KCl, 138 mM NaCl, 0.05% Tween 20) to remove the unbound fraction. The day before the assay, streptavidin 384-well black plate (Greiner) was incubated overnight at 4°C with 20 μL of 1.5 ng/μL biotin-TUBEs in PBS to allow binding of the TUBEs to the plate. Then, plates were washed with TBS-Tween and 10 μL of lysis buffer (50 mM Tris pH 7.5, 5 mM EDTA, 150 mM NaCl, 1% Tween 20, 50 μM DUBI, complete mini-EDTA free anti-proteases, 1 mM PMSF) was added. The 384-plates prepared previously (see above) that contained iRBCs incubated with compounds were thawed and 10 μL of the culture was transferred to the streptavidin plates containing TUBEs and lysis buffer. iRBCs lysis was performed applying three freeze/thaw cycles (10 min each) and then these streptavidin plates were incubated for 1 hr at room temperature in a microtitre-plate shaker to favour the capture of all the ubiquitylated proteins by the TUBEs. After washing the plates to remove the unbound proteins, 10 μL of antibody anti-ubiquitin FK2 diluted 1:200 in DELFIA assay buffer (50 mM Tris HCl 7.5, 20 μM EDTA, 500 mM NaCl, 0.01% Tween 20 and 0.1% BSA) was added and incubated for 1 hr at room temperature under low shaking. Then plates were washed and an additional incubation with 10 μL of DELFIA secondary antibody (diluted 1:200 in DELFIA assay buffer) was performed for 1 hr, followed by the last washing cycles. Finally, 25 μL of the enhancement solution was added, which causes the release of the europium. Plates were then incubated for at least 15 min at room temperature with shaking before reading. Fluorescence signal from europium was recorded in the Envision plate reader using a protocol for time-resolved fluorescence with excitation at 340 nm and emission at 615 nm. The long fluorescence lifetime of europium enables the use of time-resolved fluorimetry to eliminate background interferences.

### Data analysis

The effect of compounds was calculated taking as reference the basal level of ubiquitylated proteins in iRBCs (control 1 = basal levels of ubiquitylated proteins in cell lysates in 1% DMSO, in the absence of compound). The increase in the levels of the ubiquitylated proteins was calculated using the stimulation formula: % stimulation = 100*(sample/control 1), being 100% the basal levels without stimulation (control 1). Compounds that inhibit the proteasome activity will cause an increase in the levels of ubiquitylated proteins, and hence an increase in the AlphaLISA or DELFIA signals. Samples above the cut-off (calculated as the average of control 1 plus three times its standard deviation) were considered positives. TIBCO Spotfire 3.2 software was used to perform this analysis.

In the dose response graphs, all curves were normalized between 0 and 100% inhibition. Maximum levels of ubiquitylated protein for each compound were considered as 100% of UPS inhibition (B). Minimum levels of ubiquitylated proteins (0%) were the basal levels without any treatment (A). Data were calculated normalizing the raw counts with respect to the minimum (A) and maximum (B) counts per compound (% inh = 100 – (100 * (sample – B)/(A – B)). pIC50s (−log molar IC50) were calculated and represented using GraFit 5 (Erithacus Software, Horley, Surrey, UK).

## Results

### Assessment of the levels of ubiquitylated proteins at different parasite stages

*Plasmodium falciparum* intra-erythrocytic cycle, which includes rings, trophozoites and schizonts stages (Figure 1A) was used to set up the assays as it is related with its pathogenesis and is the primary target of anti-malarial drug development. Total amount of ubiquitylated proteins at each stage of the intra-erythrocytic cycle was analysed by Western blot using anti-ubiquitin P4D1 antibody. Figure 1B shows the levels of ubiquitylated proteins in parasite extracts (see Methods – “*Plasmodium falciparum* extracts for assay development) during ring, trophozoite and schizont stages. The levels of ubiquitylated proteins increased along the cycle, reaching the highest concentration in schizonts in accordance with previously reported data [9].

To determine the stage containing the largest accumulation of ubiquitylated proteins after treatment with a UPS inhibitor, two different proteasome inhibitors were used: the well-known MG132 and epoxomicin. iRBCs with *P. falciparum* at different stages were treated for 1 hr with these proteasome inhibitors. Atovaquone was used as negative control, as its mode of action is not related to UPS. *Plasmodium falciparum* proteins were isolated and then quantified by Bradford to normalize the amount of proteins loaded in the gel. As expected, atovaquone did not change the amount of ubiquitylated proteins while MG132 and epoxomicin treatments resulted in an accumulation of ubiquitylated proteins at schizont stage of 1.8 and 1.5 times, respectively, when compared to the basal levels (Figure 1C), correlating with previously published data [9]. Taking into account these results, parasites at schizont stage were used for further assays.

### Set-up of a homogenous AlphaLISA assay to measure the levels of ubiquitylated proteins from cell lysates

The aim was to establish a universal assay to measure the total amount of ubiquitylated proteins from cells, under native conditions, in a HTS format and based on ubiquitin-traps (TUBEs) that specifically capture and protect ubiquitylated proteins with high affinity. To set up this biological assay, *P. falciparum* iRBCs were used with the added challenge of dealing with the abundant haemoglobin present in the host cells.

**Figure 2:**
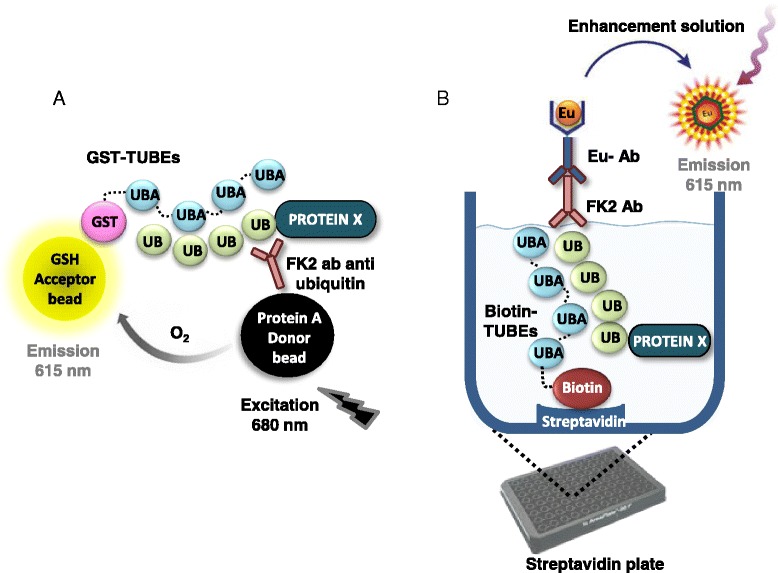
Schematic representation of the developed assays **A**. *TUBE-AlphaLISA assay.* Ubiquitylated proteins from cell lysates are captured by the GST-TUBE trap during the lysis. The GST-TUBEs trap binds to GSH AlphaLISA acceptor beads. FK2 antibody recognizes and binds to ubiquitin chains covalently attached to substrates and it is recognized by the protein A AlphaLISA donor bead. AlphaLISA donor bead is excited at 680 nm releasing singlet oxygen that diffuses towards the acceptor bead. After excitation acceptor bead emits light at 615 nm. **B**. *Schematic representation of TUBE-DELFIA assay* **.** *P. falciparum* iRBCs are lysed in presence of biotin-TUBEs attached to streptavidin coated plates. Ubiquitylated proteins from cell lysates captured by the TUBEs ubiquitin traps are then immunodetected by the anti-ubiquitin FK2 mouse antibody which is recognized by a secondary antimouse antibody labeled with europium. The addition of the enhancement solution causes the release of the europium generating signal at 615 nm. Washes are required before each incubation step to remove unbound proteins/reagents.

Initially, the TR-FRET technology was tested using many combinations of tagged-TUBEs, antibodies and fluorophores but results were not successful, maybe due to the large distance between the fluorophore pair. Different combinations of antibodies, beads and tagged TUBEs were also tested using the AlphaLISA technology, which enables the detection of larger complexes than TR-FRET. Only one combination among tens tested was successful, which is indicated in Figure 2A. All these failed attempts reflect the difficulty of establishing this method to detect levels of ubiquitylated proteins in cell lysates, with the added difficulty of the presence of interfering haemoglobin. Glutathione present on AlphaLISA acceptor beads recognizes the GST-tag of the TUBEs, which tightly interact with ubiquitylated proteins present in cell extracts. These ubiquitin moieties covalently linked to the proteins are also recognized by the ubiquitin antibody FK2. Protein A AlphaLISA donor beads bind to Fc domain of this anti-ubiquitin antibody. Therefore, only in presence of ubiquitylated proteins of the extracts AlphaLISA beads are close enough to enable the energy transfer. The excitation of the protein A donor beads by a laser beam at 680 nm produces the release and diffusion of singlet oxygen molecules that react with a thioxene derivative in the acceptor beads, generating chemiluminescence at 370 nm. This reaction further activates fluorophores present on the same bead that subsequently emit light, which can be detected at 615 nm. The signal emitted by the acceptor beads is proportional to the amount of polyubiquitin chains present in the cells. Singlet oxygen falls to the ground state and no signal is produced in absence of the complex.

**Figure 3:**
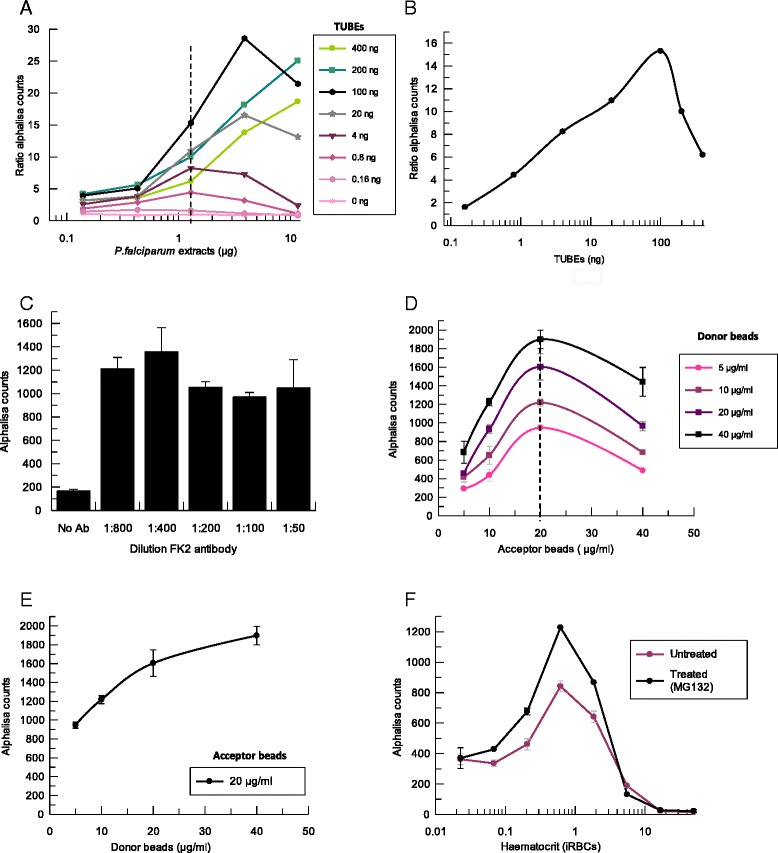
Optimization of the components of the AlphaLISA assay. Different panels show the titration of all the reagents. X axis of A, B and F panels are represented in logarithmic scale **A**. Cross titration of TUBEs and ubiquitylated proteins present in *P. falciparum* cell extracts, using 10 μg/mL of beads and 1:200 FK2 antibody dilution. Signal is represented as the ratio of AlphaLISA counts in presence an absence of TUBEs. **B**. Concentration curve of TUBEs at 1.3 μg of *P. falciparum* cell extracts. **C**. Titration of the anti-ubiquitin FK2 antibody with 100 ng TUBEs, 1.3 μg *P. falciparum* cell extracts and 10 μg/mL of beads. **D**. Cross titration of AlphaLISA donor and acceptor beads at conditions previously determined (100 ng TUBEs, 1:400 FK2 antibody and 1.3 μg of *P. falciparum* cell extracts). **E**. Titration of donor beads at 20 μg/mL of acceptor beads. **F**. iRBCs with schizonts at 100% parasitaemia and adjusted to different haematocrits with and without treatment of MG132.

In order to determine the optimal amount of each component, proteins from cell extracts, TUBEs, antibody, and beads were titrated, keeping all other components fixed. BSA (0.1%) was added to the AlphaLISA assay buffer and Triton X-100 to the lysis buffer to decrease the non-specific binding. DTT (1.5 mM) was also added to keep the glutathione reduced and optimize glutathione/GST-TUBEs recognition. To avoid interferences coming from the haemoglobin present in RBCs, during the assay development the source of ubiquitylated proteins came from isolated and lysed *P. falciparum* parasites (see Methods, “Purification of iRBCs with schizonts and treatment with compounds”). The determination of the optimal amount for each reagent is shown in Figure 3. Figure 3A shows a cross-titration of TUBEs and *P. falciparum* cell extracts. Beads concentration was fixed at 10 μg/mL and FK2 antibody was used at 1:200. Signal was normalized with respect to the basal level obtained in each condition. No signal was detected in absence of TUBEs or *P. falciparum* cell extracts. AlphaLISA counts were directly dependent on both components as expected, reaching a maximum at the optimal concentration of 100 ng of TUBEs. *Plasmodium falciparum* cell extracts at 1.3 μg/well were chosen for subsequent assays because at this concentration the system is able to detect both increased or decreased amounts of ubiquitylated proteins within the linear range (0.4-4 μg/well). Figure 3B shows the concentration curve for TUBEs at fixed amount of 1.3 μg of *P. falciparum* cell extracts. Beyond the optimal concentrations stated before, a decrease in the signal was observed due to a ‘Hook effect’, a common phenomenon found in AlphaLISA assays. The Hook effect occurs when beads are saturated with analyte. Thus, excess of analyte disrupts associations between donor and acceptor beads, causing a progressive decrease in the signal.

Next, we studied the effect of the antibody concentration on the assay using 10 μg/mL of beads and the conditions previously established for TUBEs (100 ng) and *P. falciparum* cell extracts (1.3 μg) (Figure 3C) were studied. Maximum signal of the system was reached when antibody was used at the optimal dilution 1:400. Finally, a titration of AlphaLISA acceptor and donor beads was performed to determine their optimal concentrations (Figure 3D). A Hook effect was observed again, showing a maximum signal at 20 μg/mL of acceptor beads for any donor beads concentration. Figure 3E shows the titration of the donor beads with 20 μg/mL of acceptor beads. Beads concentration for further experiments was fixed at 20 μg/mL acceptor beads and 40 μg/mL donor beads.

As explained above, the optimal concentration of each component was established using isolated *P. falciparum* cell extracts, that is, lysed parasites in which haemoglobin was washed off to avoid interferences in the read-out. As the final objective was to develop an assay sensitive enough to detect changes in the level of the total parasite ubiquitylated proteins inside the iRBCs, the optimum haematocrit (percentage of the volume of RBCs *versus* total volume) for *P. falciparum* iRBCs at 100% parasitaemia was determined, as well as the assay sensitivity in presence of an UPS inhibitor. Figure 3F shows iRBCs with schizonts at 100% parasitaemia, treated for 1 hr with the proteasome inhibitor MG132 at 1.5 μM. These iRBCs prepared at different haematocrits were added to the plates and then levels of ubiquitylated proteins were measured with the AlphaLISA assay using the conditions previously established. A range of haematocrit between 0.2 and 2% generated an acceptable signal compared to background (ratio of accumulated ubiquitylated proteins in presence of MG132 *versus* basal levels) despite haemoglobin interferences with the AlphaLISA signal. Thus, haematocrit 0.4% of purified iRBCs with schizonts was chosen for further AlphaLISA assays, as this concentration was within the linear range and allow the detection of an increased amount of proteins in presence of the proteasome inhibitor MG132.

Besides the optimization described above, other critical protocol settings, such as order of addition, binding, incubation time of antibodies and beads, procedures for cell lysis in the 384- and 1,536-well plates, temperature and volume of different components of the assay were analysed and included in the final protocol described in Methods. The assay was adapted to 384- and 1,536-well plate format obtaining a similar signal to background ratio. The 1,536-well format was chosen for future HTS campaigns whose results will be published elsewhere.

### Heterogeneous DELFIA assay to measure the levels of ubiquitylated proteins from cell extracts

AlphaLISA technology is quite similar to an enzyme-linked immunosorbent assay (ELISA). It is a selective, sensitive and versatile technique and has the additional advantage that it allows performing assays in high-throughput format since washing steps are not required. However, the absence of washing steps also favours interferences that can appear in the screening of large collections of compounds, producing false positive or negative signals. A confirmatory assay to measure the levels of ubiquitylated proteins is desirable to identify possible interferences of the previous AlphaLISA assay or if high throughput is not required. Therefore, an orthogonal ubiquitin ELISA assay based on TUBEs and DELFIA technology has been established. DELFIA assay is a heterogeneous method that requires washing steps after each addition to remove unbound reagents. Advantages and disadvantages of these AlphaLISA and DELFIA assays are reflected in Table 1.

**Table 1:** **Advantages and disadvantages of these new AlphaLISA and DELFIA assays**

	**Advantages**	**Disadvantages**
**AlphaLISA**	• High throughput:	• Prone to interferences: aggregating compounds (false positive), chemical or colour quenchers (false negative)
	- Homogeneous: mix and measure assay eliminating washing steps	
	- Save time and work	
	- 1,536 well plate format	
**DELFIA**	• Lower interference:	• Lower throughput:
	- Compounds and non-specific assay reagents are removed in the washing steps	- Heterogeneous. Washes are needed: labour intensive
		- Transfer step: iRBCs transfer to streptavidin plates is required after treatment. Time consuming.
		- More sources of assay variability
		- 384-well plates

DELFIA assay was developed using biotin-tagged TUBEs to anchor ubiquitin-traps to the 384-well, streptavidin-coated plates. After treatment with the compounds, cells are plated and lysed in the presence of TUBEs, which recognizes ubiquitylated proteins with high affinity and prevents proteasome-mediated proteolysis or DUB-mediated deconjugation processes. Anti-ubiquitin FK2 antibody binds ubiquitylated proteins and it is recognized by an anti-mouse DELFIA antibody labelled with europium (Figure 2B). This DELFIA sandwich is then treated with enhancement solution to release europium from the secondary antibody, producing signal at 615 nm that is proportional to the amount of ubiquitylated proteins present in the well.

**Figure 4:**
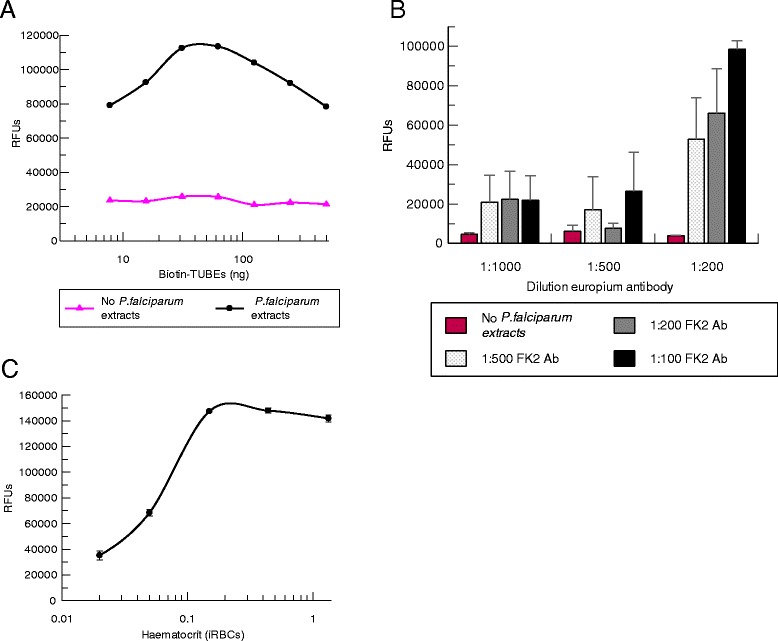
Optimization of the components of the TUBE-DELFIA assay. X axis of panels A and C are represented in logarithmic scale. **A**. Titration of TUBEs with saturating concentration of proteins from isolated *P. falciparum* cell extracts (10 μg), using anti-ubiquitin FK2 and europium antibodies at 1:200 dilution. **B**. TUBE-DELFIA assay using a saturating concentration of proteins from isolated *P. falciparum* cell extracts (10 μg), 30 ng TUBEs and three different concentrations of anti-ubiquitin FK2 and secondary europium antibodies. A control without proteins was used to determine the background signal. **C**. Haematocrit titration of iRBCs with schizonts at 100% parasitaemia treated with MG132 inhibitor (1.5 μM), using the conditions previously established (30 ng TUBEs, 1:100 FK2 and 1:200 europium antibodies).

Each component of the assay was optimized in a similar way as described for AlphaLISA assay (Figure 4). The first component evaluated was the biotin-TUBEs, which were titrated-fixing concentrations for primary and secondary antibodies at 1:200 in absence or presence of saturating concentration of proteins from isolated *P. falciparum* cell extracts (10 μg). BSA (0.1%) was also added to the antibody solutions to decrease the non-specific binding. Saturation of the streptavidin-coated well was reached at 30 ng of biotin-TUBEs. This amount of biotin-TUBEs was used in subsequent experiments as concentrations higher than 60 ng produced a decrease in the signal which was specific and dependent. Signal was specific and dependent on the presence of TUBEs and ubiquitylated proteins because no changes in signal were observed in the absence of cell extracts (Figure 4A). In order to determine the optimal dilution of primary and secondary antibodies, they were crosstitrated using three different dilutions. The assay was also performed in the absence of *P. falciparum* proteins to estimate the background signal. Dilutions 1:200 of europium antibody and 1:100 of FK2 primary antibody were chosen for further set up experiments as they produced the highest signal to background ratio (Figure 4B).

Once TUBEs and antibody dilutions were optimized, the haematocrit of the culture that displayed the maximum signal was established by testing different amounts of *P. falciparum* iRBCs at 100% parasitaemia (Figure 4C). Cultures at different haematocrits were treated for 1 hr with the proteasome inhibitor MG132 (1.5 μM) to allow the maximal accumulation of ubiquitylated proteins and saturation of TUBEs. Treatment was performed in another plate to avoid interferences with the streptavidin-coated plates. After the incubation with the inhibitor, iRBCs were transferred to the streptavidin plate previously coated with biotin-TUBEs. Maximal signal was observed with 0.2% haematocrit. Concentrations above 0.2% did not produce changes in the signal because of saturation of the TUBE binding sites.

### Pharmacological validation of AlphaLISA and DELFIA assays using known proteasome inhibitors

**Figure 5:**
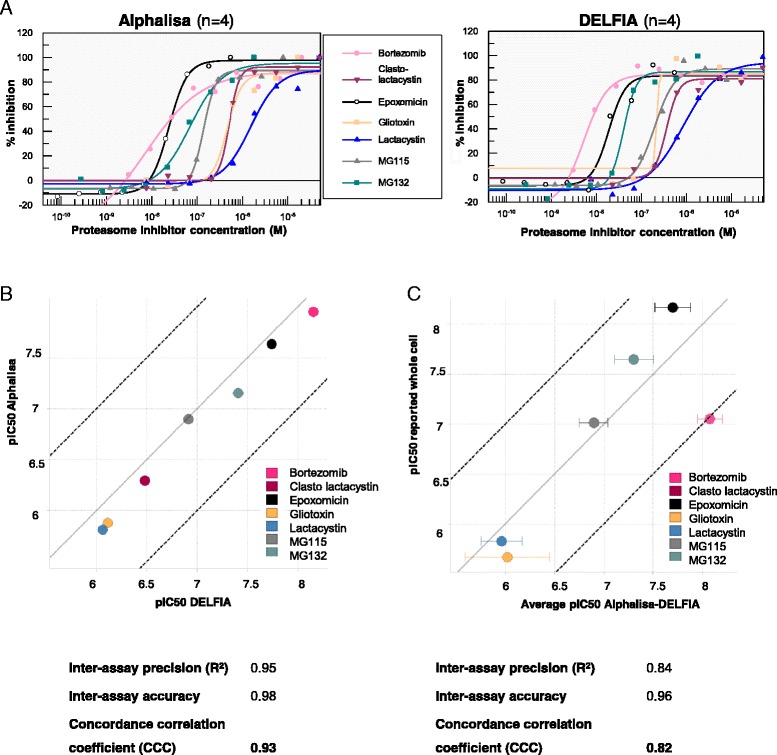
Correlation between AlphaLISA and DELFIA assays. **A**. Dose–response curves of known proteasome inhibitors in TUBE-AlphaLISA (left panel) and TUBE-DELFIA assays (right panel). The response is represented as a percentage of inhibition, which is calculated normalizing the data to the maximal signal obtained for each compound (see data analysis section). **B**. Correlation of pIC50s in TUBE-AlphaLISA and TUBE-DELFIA assays (average of at least 2 copies). Each point corresponds to a particular proteasome inhibitor. Interassay accuracy, Pearson’s correlation and CCC (the multiplication of both values) are shown. Grey line represents perfect correlation (y = x) while black doted lines are the perfect correlation ± 1 log unit (y = x ± 1). **C**. Correlation of the average of pIC50 obtained in the AlphaLISA and DELFIA assays *vs* reported pIC50 that produce the parasite growth inhibition in the whole cell assay.

Assays developed in HTS format for drug discovery should first be validated for biological and pharmacological relevance but also for assay performance. Seven different proteasome inhibitors, known to inhibit the growth of *P. falciparum* cells [14], were tested at different concentrations in both assays to verify that they produced an accumulation of ubiquitylated proteins and that the assays were sensitive enough to detect this increment in a robust manner. iRBCs with *P. falciparum* schizonts at 100% parasitaemia were obtained as described in Methods and treated for 1 hr with compounds. Dose–response experiments were performed starting at different concentrations according to the reported IC50 of each compound [14]. For representation purposes, compound effects have been normalized between 0 and 100% inhibition, the maximum (100%) being the highest amount of accumulated ubiquitylated proteins for each inhibitor and the minimum (0%) being the basal level of ubiquitylated proteins without inhibitor (Figure 5A).

Proteasome inhibition caused by the compounds tested, led to an accumulation of *P. falciparum* ubiquitylated proteins in a dose-dependent manner. Bell effect was observed with some compounds at the highest concentrations tested, but it was not related with the saturation of the assay components (Hook effect) as it was observed at different levels of maximum response. This effect could be due to the biological mode of action of the compound. Point of curve showing this effect was not included in the IC50 fitting. Assays with uRBCs at the same haematocrit were performed in parallel to confirm that observed changes in ubiquitylated protein levels occurred in the parasite and not in the host. No significant changes were observed in the presence of proteasome inhibitors, as the host proteasome is not very active.

The intrinsic variability of the assay is reflected in the different maximal responses obtained with these tool compounds in different replicates that correspond to different days (Table 2), although this fact did not affect the pIC50 values (Figure 5B). Maximum stimulation reached for each compound was in the range of 1.3-2.7-fold (Table 2) regarding basal levels. Factors contributing to this intrinsic variability could be due to synchronization and purification steps of the cells as well as the parasite biology. Results also indicate that a compound with a pIC50 between 5.6-8.2 in whole cell, acting upon the UPS, can be picked up with these two assays.

**Table 2:** **pIC50 and maximum percentage of stimulation of proteasome inhibitors in different assays**

**Proteasome inhibitor**	**pIC50 reported whole cell**	**pIC50 AlphaLISA (n = 4)**	**Maximum% stimulation AlphaLISA**	**pIC50 DELFIA (n = 4)**	**Maximum% stimulation DELFIA**
Epoxomicin	8.17	7.64 ± 0.09	178 ± 47	7.74 ± 0.24	177 ± 15
Bortezomib	7.05	7.95 ± 0.08	178 ± 24	8.16 ± 0.05	180 ± 14
Gliotoxin	5.66	5.88 ± 0.73	217 ± 53	6.12 ± 0.27	180 ± 26
MG132	7.65	7.17 ± 0.04	236 ± 36	7.41 ± 0.21	189 ± 55
MG115	7.01	6.88 ± 0.09	208 ± 22	6.92 ± 0.21	185 ± 17
Lactacystin	5.83	5.81 ± 0.07	217 ± 27	6.06 ± 0.22	193 ± 32
Clasto-lactacystin β lactone	5.83	6.30 ± 0.09	195 ± 19	6.48 ± 0.18	198 ± 17

The correlation of pIC50s from both assays is shown in Figure 5B. pIC50 is the negative logarithm of the concentration in molar units that produces the 50% of the biological response (pIC50 = −log [IC50]), in this case, the increase of ubiquitylated proteins. The inter-assay accuracy, which is the measure of the deviation of the assay average from one assay to the other under perfect match conditions, as well as the precision (Pearson’s correlation), was estimated for each assay. Thus, the concordance correlation coefficient (CCC) that takes into account both parameters (CCC = interassay precision × interassay accuracy) was calculated for DELFIA *vs* AlphaLISA assays, obtaining a value of 0.93, which means that the match between both assays is perfect. Assays with values of CCC greater than 0.81 are considered identical (perfect match) as CCC is the product of two correlation values.

pIC50 average of the compounds in both assays was also compared with the reported pIC50 that led to the parasite growth inhibition in the whole cell assay [14] (Figure 5C and Table 2). The pIC50 of bortezomib in whole-cell assay is the average of the pIC50 previously reported [13,14] as both published data differ in 0.91 log units. The CCC obtained was 0.82, which is a perfect match even when pIC50s were obtained with one hour of compound incubation, while the whole-cell assay requires at least 48 hours of incubation (Figure 5C).

These results strongly suggest that the UPS is the primary target of these compounds and that the parasite death is due to its deregulation. It confirms the relevance of the UPS as a drug target for malaria treatment and also validates these AlphaLISA and DELFIA assays to detect UPS inhibitors in HTS conditions.

### Assay robustness

**Figure 6:**
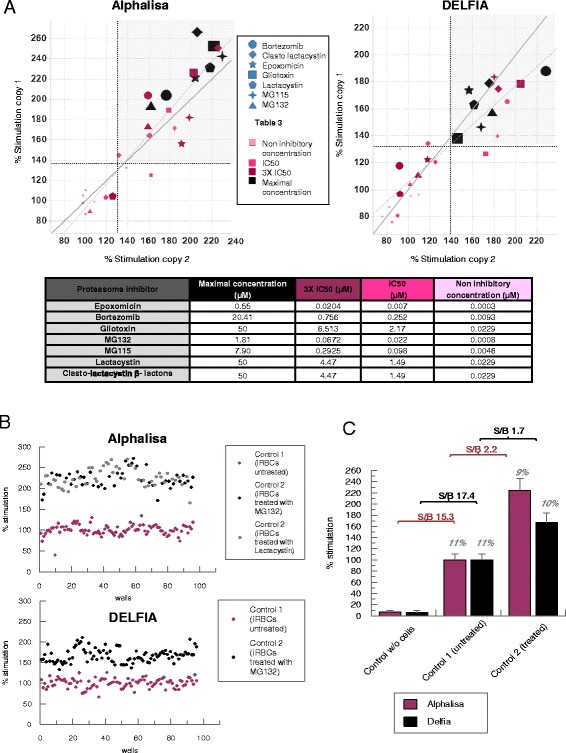
Assay robustness and validation. **A**. Correlation of two plates with proteasome inhibitors located in known positions at four different concentrations simulating a screening plate (see embedded Table). Response produced by proteasome inhibitors on the levels of ubiquitylated proteins represented as % of stimulation (% of stimulation = 100*(sample/average control 1). The size of the points is proportional to the compound concentration tested. Light grey is the linear regression of the data while dark grey is the perfect correlation (y = x). Black dotted lines represent the statistical cut-off of each replicate (average control 1 + 3*standard deviation). Positive hits are above the cut off. **B**. Graph of control 1 (iRBCs without treatment), and control 2 (iRBCs with proteasome inhibitor 1.5 μM MG132 or 15 μM lactacystin) in the screening plates (n = 96 wells) represented as % of stimulation. **C**. Comparison of AlphaLISA (purple) and DELFIA (black) assay control data: robust average in bars, robust CV in italics and S/B in bold for control 1, control 2 and control without lysates in both assays.

The suitability of both assays to carry out HTS campaigns was determined with previously tested proteasome inhibitors scattered in known positions within the plates (spike plates) to simulate a real HTS compound plate. They were placed at four different concentrations: a) the maximum concentration of each curve in dose response assays (Figure 5A); b) three times the reported IC50 (whole cell); c) the reported IC50 (whole cell); and, d) a non-inhibitory concentration. In Figure 6A, size of the points for each compound is proportional to the concentration tested (see embedded Table). Bigger symbols indicate higher concentrations. The level of ubiquitylated proteins present in *P. falciparum* iRBCs with schizonts was calculated as a percentage of stimulation compared with ubiquitylated protein basal levels (control 1 without any treatment), which corresponds to 100% of stimulation. A positive control of stimulation (control 2) using two known proteasome inhibitors, MG132 (1.5 μM) or lactacystin (15 μM), was also included for internal reference. Both caused similar stimulation levels (calculated as an average of all points with either of these two inhibitors). Compounds whose response was above the statistical cut-off (calculated as the average of the control 1 population plus three times the standard deviation), which is around 30%, were considered as positive in the assays. Some compounds showed the previously mentioned bell effect, such as gliotoxin and lactacystin in DELFIA assay, and also clasto-lactacystin-β-lactone and MG132 in AlphaLISA assay (Figure 6A). Despite that, results indicate that it is possible to find inhibitors in a screening plate tested at 10 μM with either assay, as the percentage of stimulation of all the compounds is above the statistical cut-off at that concentration.

Percentage of stimulation of iRBCs treated with the proteasome inhibitor MG132 or lactacystin (control 2) as well as of iRBCs without treatment (control 1), is represented in Figure 6B. These results were obtained from 96 different wells for each control and show that the robustness of the assays’ performance making them suitable for screening campaigns. In Figure 6C bars represent the robust average of control 1 (basal levels of ubiquitylated proteins, no treatment), control 2 (cells treated with MG132 or lactacystin) and a control without *P. falciparum* cell lysates in both assays. Control 1 was 100% of stimulation while control 2 was around 220% in AlphaLISA and 170% in DELFIA assay. Robust coefficient of variation (CV), which is calculated with the formula CV = 100*(robust standard deviation/robust average), was similar in both assays. CV values were below 15% in both cases, which is the maximum permissible limit to consider a stimulation assay robust. Due to the low signal to background obtained with these tool compounds and the maximal asymptote variability, it is recommended not to use the control 2 to normalize the data in a HTS campaign, but to include it as a positive control of stimulation in all the plates. Signal-to-background ratio (S/B) between basal levels of ubiquitylated proteins and the signal obtained without cell extracts was 15.3 and 17.4 in AlphaLISA and DELFIA, respectively. Altogether these results confirm that both assays are able to differentiate levels of ubiquitylated proteins and that the small window between control 1 and control 2 is due to small differences of total ubiquitylated proteins in the presence of proteasome inhibitors (Figure 1C). Despite that, a screening campaign with both assays is ongoing and reproducible results are being obtained.

## Discussion

After phenotypic screening efforts in the malaria field, many inhibitors have been found with good *in vitro* potencies in growth inhibition assays [31-33]. The mode of action of all these hits is unknown. Assays able to disclose the target or, at least, the pathway they are hitting are urgently needed to focus on novel mechanisms of action to overcome emerging resistance. For this reason, two universal assays have been developed that are based on AlphaLISA and DELFIA technologies, coupled to TUBEs traps, to identify compounds that modify the activity of the UPS within the cell, as no current anti-malarial treatment is targeting this pathway. These assays are based on the accumulation of ubiquitylated proteins after compound treatments, making them ideal for broad applications. The assays could also be applied to a large diversity of cell lines and pathologies, in which the UPS plays an essential role, such as cancer, inflammatory diseases, etc.

Working with 1,536-well plates and RBCs had two added challenges in the development of the assays. First, no lysis procedure was previously published in this type of plate, so a new protocol was set up for this critical step. And, the second challenge was the presence of haemoblogin from the RBC, as its presence interfered with AlphaLISA assay. Despite this, the S/B with regard to the sample without RBCs was around 15-fold in AlphaLISA assay. This S/B is very similar to that obtained in DELFIA, where no haemoglobin is present during the read-out, as it is fully washed away. Both assay formats have been able to detect changes in the levels of ubiquitylated proteins in presence of different proteasome inhibitors, obtaining pIC50s that perfectly correlate with the known pIC50 that cause parasite growth inhibition [14]. A short incubation time of just one hour in the assays, allows the conclusion that UPS is the primary target of the tested compounds, the UPS deregulation being the principal cause of the parasite death.

These AlphaLISA and DELFIA assays can be used in HTS assay conditions as known proteasome inhibitors located in random positions in a screening plate have been detected at different concentrations, all of them being above the statistical cut-off at 10 μM. Moreover, the CV of the control was lower than 15% showing their robustness. AlphaLISA is a more suitable assay to perform HTS of large compound collections as it has been adapted to 1,536-well plate format, while DELFIA assay would be more useful as an orthogonal assay to confirm, in dose response, primary hits detected in AlphaLISA assay. Source of interferences in DELFIA are much lower than in AlphaLISA because unbound reagents are removed after washes and compounds are not present during the binding of the different components. Nonetheless, if a small number of compounds are screened, TUBE-DELFIA assay can be used as the primary option.

Since total ubiquitylation is assessed from living cells cultured *in vitro* in both assays, all kinds of cell-penetrating UPS inhibitors or activators that affect ubiquitylated proteins levels within the cell could be detected. The most obvious compounds could be those having an effect on the proteasomal activity as it controls a higher number of ubiquitylated proteins than other components of the UPS. Compounds affecting DUBs or E3 ligases with a broad spectrum of substrate proteins could also be identified, being a promising source of selective compounds [12] However, the majority of E3 ligases and DUBs exhibit substrate specificity making it more difficult to identify compounds that affect the levels of ubiquitylation of a small number of proteins with the assays described. To discover the precise target, biochemical assays are commercially available for testing proteasome or DUBs inhibition.

The beauty of this approach is that extensive previous knowledge about the target pathway is not required to find inhibitors, as compounds blocking any step of the UPS that produce a significant variation in the pool of ubiquitylated proteins can be found. The target deconvolution of the compounds obtained through this approach could also lead to identify new druggable targets within the UPS.

## Conclusions

AlphaLISA and DELFIA assays developed here can be considered as universal systems to assess changes in the total ubiquitylated protein levels and could be adapted to detect specific ubiquitylated proteins just by changing the primary antibody. Here, whole ubiquitilome changes in *P. falciparum* have been measured using TUBEs and anti-ubiquitin FK2 antibody, but this antibody could be changed for any other against the protein of interest. Thus, activity of a specific DUB or ligase can be assayed in HTS campaigns in whole cells and under native conditions without using recombinant proteins or engineered cells. Applications using these assays can be numerous, not being necessarily related with compound identification, but with the biology of a certain process or protein. The quantification of the levels of the specific components or the whole UPS can also be useful to investigate the molecular basis of different diseases as they can be adapted to study any other pathology in which the UPS plays an essential role, using different cell lines and/or to measure a specific activity of some of the components of the UPS in a high throughput format.

## Abbreviations

UPS: Ubiquitin proteasome system
DUB: Deubiquitylase
DUBI: DUB inhibitor
TUBEs: Tandem ubiquitin binding entities
UBA: Ubiquitin-associated domains
iRBCs: *P. falciparum*-infected red blood cells
uRBCs: Uninfected red blood cells
PHGH: Peptidyl-glutamyl peptide-hydrolyzing
HTS: High-throughput screening
GST: Glutathione S-tranferase
ELISA: Enzyme-linked immunosorbent assay
S/B: Signal to background
CV: Coefficient of variation
PMSF: Phenylmethylsulphonyl fluoride
DTT: Dithiothreitol
GSH: Glutathione acceptor beads
BSA: Bovine serum albumin
TR-FRET: Time-resolved fluorescence resonance energy transfer
DELFIA: Dissociation-enhanced lanthanide fluorescent immunoassa
ECL: Enhanced chemiluminescence
PBS: Phosphate buffer solution
PVDF: Polyvinylidene difluoride
CCC: Concordance correlation coefficient
